# Complications and mortality of venovenous extracorporeal membrane oxygenation in the treatment of neonatal respiratory failure: a systematic review and meta-analysis

**DOI:** 10.1186/s12890-020-1144-8

**Published:** 2020-05-07

**Authors:** Jing Xiong, Li Zhang, Lei Bao

**Affiliations:** 1 Department of Neonatology, Ministry of Education Key Laboratory of Child Development and Disorders, National Clinical Research Center for Child Health and Disorders, China International Science and Technology Cooperation base of Child development and Critical Disorders, Children’s Hospital of Chongqing Medical University, Chongqing Key Laboratory of Pediatrics, Chongqing, People’s Republic of China; 2 Department of Pulmonology, Children’s Hospital of Chongqing Medical University, No.136, Zhongshan second road, Yuzhong district, Chongqing, 400014 China

**Keywords:** Extracorporeal membrane oxygenation, Respiratory failure, Systematic reviews, Meta-analysis, Neonate

## Abstract

**Background:**

Extracorporeal membrane oxygenation (ECMO) has been increasingly used for severe neonatal respiratory failure refractory to conventional treatments. To systematically evaluate the complications and mortality of venovenous ECMO (VV ECMO) in the treatment of neonatal respiratory failure, we performed a systematic review and meta-analysis of all the related studies.

**Methods:**

PubMed, Embase, and Cochrane Library were searched. The retrieval period was from the establishment of the database to February 2019. Two investigators independently screened articles according to the inclusion and exclusion criteria. The quality of article was assessed by the Newcastle-Ottawa scale (NOS). The meta-analysis was performed by Stata 15.0 software.

**Results:**

Four observational studies were included, with a total of 347 newborns. VV ECMO was used for neonates with refractory respiratory failure unresponsive to maximal medical therapy. Median ages of the newborns at cannulation were 43.2 h, 23 h, 19 h, and 71 h in the included four studies, respectively. The overall mortality at hospital charge was 12% (5–18%) with a heterogeneity of I^2^ = 73.8% (*p* = 0.01). Two studies reported mortality during ECMO and after decannulation, with 10% (0.8–19.2%) and 6.1% (2.6–9.6%), respectively. The most common complications associated with VV ECMO were: pneumothorax (20.6%), hypertension (20.4%), cannula dysfunction (20.2%), seizure (14.9%), renal failure requiring hemofiltration (14.7%), infectious complications (10.3%), thrombi (7.4%), intracranial hemorrhage or infarction (6.6%), hemolysis (5.3%), cannula site bleeding (4.4%), gastrointestinal bleeding (3.7%), oxygenator failure (2.8%), other bleeding events (2.8%), brain death (1.9%), and myocardial stun (0.9%).

**Conclusion:**

The overall mortality at discharge of VV ECMO in the treatment of neonatal respiratory failure was 12%. Although complications are frequent, the survival rate during hospitalization is still high. Further larger samples, and higher quality of randomized controlled trials (RCTs) are needed to clarify the efficacy and safety of this technique in the treatment of neonatal respiratory failure.

## Background

Severe neonatal respiratory failure is associated with substantial mortality [1, 2]. Despite the great development of mechanical management and some other conventional therapies, mortality is still high, and prognosis of neonates with extremely low oxygenation is especially poor [3]. Some complications such as ventilator-induced lung injury caused by mechanical ventilation may also affect the prognosis in return [4]. Extracorporeal membrane oxygenation (ECMO) is a rescue therapy for the treatment of severe neonatal respiratory failure refractory to high-frequency oscillatory ventilation (HFOV), pulmonary surfactant (PS) replacement, inhaled nitric oxide (iNO), and other conventional treatments [5–7]. Nowadays, ECMO is used to treat various reversible neonatal diseases, the most common diagnoses are meconium aspiration syndrome (MAS), persistent pulmonary hypertension of newborn (PPHN), and congenital diaphragmatic hernia (CDH) [6]. With the development of new therapies such as HFOV, exogenous surfactant therapy, and iNO, fewer patients with MAS, PPHN and RDS are supported by ECMO [8–10]. However, the survival rate of neonates with MAS has been sustained highest, approximately 94%. The survival rates of neonates with RDS and PPHN come to the next, with 84 and 77%, respectively. Whereas patients with CDH had the worst survival in this cohort of patients, approximately 51% [6]. There are two types of ECMO that are mostly used, one is venoarterial ECMO (VA ECMO) that provide both respiratory and cardic support; the other is venovenous ECMO (VV ECMO) that provide solely respiratory support. In this study, we aimed to evaluate the incidence of complications and in-hospital mortality of VV ECMO in the treatment of neonatal respiratory failure.

## Methods

### Literature search

We conducted a systematic review and meta-analysis in accordance with Meta-analysis of Observational Studies in Epidemiology (MOOSE) and the Preferred Reporting Items for Systematic Review and Meta-Analysis (PRISMA) guidelines [11, 12]. Pubmed, Embase, and Cochrane library were searched systematically for articles reporting on VV ECMO in the treatment of neonatal respiratory failure. The retrieval period was from the establishment of the database to February 2019. We used Mesh terms with the following search strategies: (“extracorporeal membrane oxygenation” OR “Oxygenators, membrane”) AND (“Adult respiratory distress syndrome” OR “Respiratory insufficiency”) AND “infant, newborn”. Language was restricted to English only. We also searched references of included articles to identify additional studies. Two investigators reviewed the citations independently.

### Selection criteria

The title and abstract of citations were screened initially and full text was reviewed with the following inclusion criteria: (a) Randomized controlled trials (RCTs) and quasi-randomized controlled trials or observational studies; (b) Neonates with respiratory failure; (c) Neonates receiving VV ECMO, if VV ECMO and VA ECMO mixed, only studies reporting on independent outcomes for each mode were included, or the percentage of VA ECMO usage rate in the study was less than 10%, which produced negligible effect on the statistical analysis. (d) Studies reporting on complications and mortality during hospitalization. (e) Neonates more than 50. Articles that met all the inclusion criteria were included. A sample size cut-off of 50 VV ECMO cases and the percentage cut-off of 10% VA ECMO cases per study were established to limit the undue influence of anecdotal cases and to minimize publication bias, in keeping with prior systematic review and meta-analysis in adults [13]. Exclusion criteria including: (a) Case report, review, conference abstract, animal experiment, systematic review, meta-analysis and so on; (b) Duplicated studies; (c) Studies registered in the extracoporeal life support organization (ELSO) database; (d) Less than 50 patients; (e) Studies without available outcomes of interest. The corresponding authors were contacted to request additional data.

### Data extraction and quality assessment

Two investigators (JX and LZ) performed data extraction independently, disagreements were settled by a third investigator (LB). The inclusion and exclusion criteria were strictly followed in the process of literature screening. The following data were collected: demographic data of patients, features of included studies, procedural details and equipment information of ECMO including maximum cannula size, pump type, oxygenator type, and cannula type. The main outcomes of interest included mortality during ECMO or at discharge and incidence of complications. We used the Newcastle-Ottawa scale (NOS) to evaluate the quality of the included studies [14].

### Statistic analysis

We used Stata ve.15.0® licensed for StataCorp, College Station, TX, USA for statistic analysis to quantitatively synthesize the mortality rate and complication rate of VV ECMO for neonates with severe respiratory failure during hospitalization. The results were presented as a summary point estimate (in %) with 95% confidential interval (CI). The heterogeneity between the studies was analyzed by the chi-square test, and was quantitatively determined by I^2^. The published guidelines quantify heterogeneity values as three levels: low (I^2^ = 25–49%), moderate (I^2^ = 50–74%), and high (I^2^ ≥ 75%) [15]. A random-effects model using DerSimonian and Laird method for variance estimator was performed to report results [16]. Statistic significance was set at a P less than 0.05 (two-tailed).

## Results

### Study selection

**Fig. 1:**
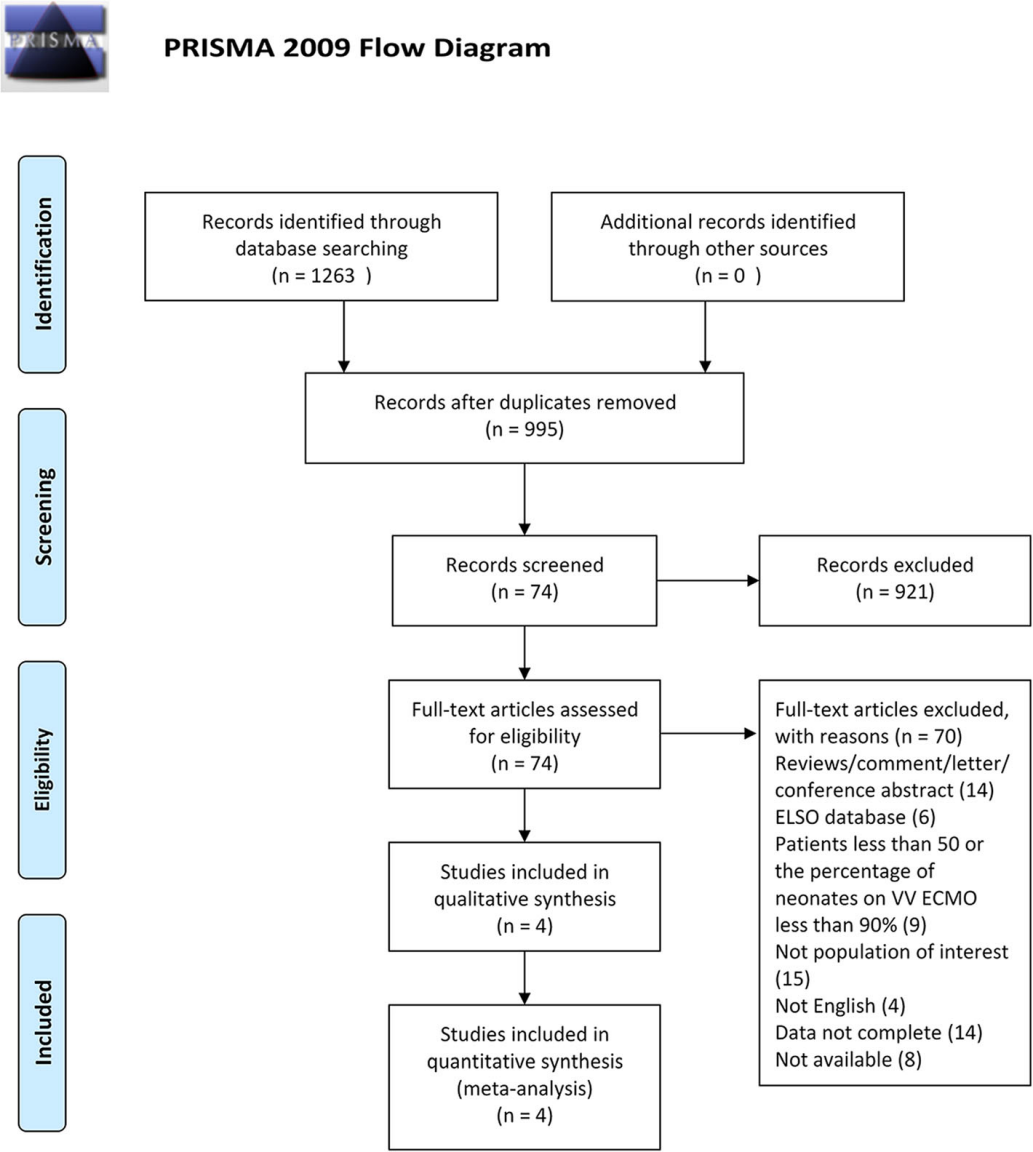
Flowchart of study screening for the systematic review and meta-analysis

One thousand two hundred sixty-three studies (564 in Pubmed, 665 in Embase, 34 in Cochrane library) were initially reviewed and 4 studies were finally included with a total of 347 patients [15–18] (Fig. 1). We excluded the studies registered in the ELSO database to avoid overlapping with studies from the original center and to diminish selective bias. All the included studies were single center or multicenter observational studies, which were implemented in Europe or the United States and published in English. NOS was used to perform quality assessment since all the studies were non-RCTs. Two studies got 6 stars [17, 19], and two studies got 8 stars [18, 20], which demonstrated a high quality for each study.

### Study characteristics

Demographic data of patients, features of included studies, procedural details and equipment information of ECMO are presented in Table 1, Table 2, and Table 3, respectively. Three single center retrospective studies and one multicenter retrospective study were found. Two studies were performed 20 years ago, when polymethylpenthene hollow fiber membrane technology was not available. All included studies reported complications and mortality of VV ECMO in the treatment of severe neonatal respiratory failure. Underlying diseases leading to respiratory failure were variable, mostly included MAS and PPHN. Three studies included only VV ECMO patients, while the remaining study included patients in combination with VV ECMO and VA ECMO. Outcomes were not reported independently in this study, but the proportion of patients received solely VV ECMO was more the 90%.

**Table 1:** Demographic data of patients in included studies

Study	Number of patients	Included disease	Gestational age (weeks)	Weight (kg)	PaO2 (mmHg)	Oxygenation index	Age (hours)
Speggiorin et al. [17]	72	Mixed	40	3.4	41.2	50	43.2
Kugelman et al. [18]	114	MAS	40.3 ± 0.1	3.48 ± 0.05	35.8 ± 1.0	60 ± 3	23
Knight et al. [20]	54	Mixed	39.6 ± 0.3	3.595 ± 0.072	38 ± 2	NA	19 ± 2
Chevalier et al. [19]	102	Mixed	38.1 ± 2.2	3.054 ± 0.62	49.5	46	71 ± 94

*MAS* Meconium aspiration syndrome, *PaO2* Partial pressure of oxygen, *NA* Not available

**Table 2:** Features of included studies and quality assessment

Study	Year	Country	Design	Primary outcome	NOS score
Speggiorin et al. [17]	2015	UK	Single center, retrospective study	Mortality and complications	6
Kugelman et al. [18]	2005	USA	Single center, retrospective cohort study	Mortality and complications	8
Knight et al. [20]	1996	USA	Multicenter, retrospective cohort study	Mortality and complications	8
Chevalier et al. [19]	1993	France	Multicenter, retrospective study	Mortality and complications	6

*NOS* Newcastle-Ottawa quality assessment scale

**Table 3:** Procedural details and equipment information of ECMO

Study	VV ECMO (%)	VA ECMO (%)	VV ECMO convert to VA ECMO	ECMO duration (hours)	Site of insertion	Maximum cannula size	Oxygenator type	Cannula type	Pump type
Speggiorin et al. [17]	100%	0	0	90.5	Right internal jugular vein	16Fr	Polymethylpentene hollow fiber membrane	Double-lumen venous cannula	Ccentrifugal pump
Kugelman et al. [18]	100%	0	2	88.5	Right internal jugular vein	14Fr	NA	Double-lumen venous cannula	NA
Knight et al. [20]	100%	0	0	114 ± 9	Right internal jugular vein	14Fr	NA	Double-lumen venous cannula	NA
Chevalier et al. [19]	95.3%	4.7%	5	117.8 ± 84	Right internal jugular vein	10Fr	NA	Double-lumen venous cannula	Non-occlusive roller pump

*VV ECMO* Venovenous extracorporeal membrane oxygenation, *VA ECMO* Venoarterial extracorporeal membrane oxygenation, *NA* Not available

**Fig. 2:**
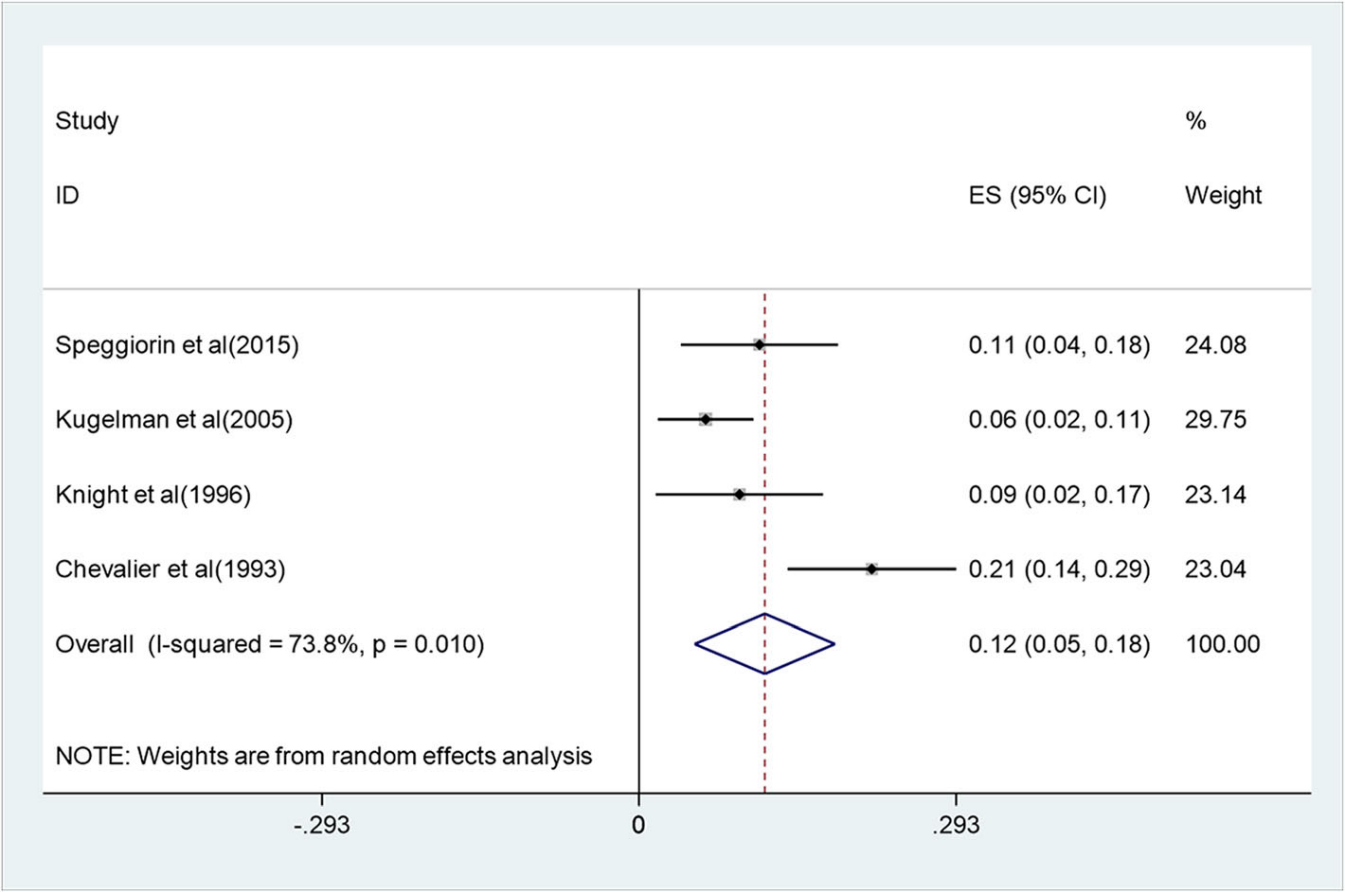
Forest plot of pooled mortality during hospitalization

Mortality at hospital discharge ranged from 6 to 21%, and pooled mortality at hospital discharge was 12% (5–18%) with a heterogeneity of I^2^ = 73.8% (*p* = 0.01) (Fig. 2). Two studies reported mortality during ECMO and after decannulation, with 10% (0.8–19.2%) and 6.1% (2.6–9.6%), respectively. Complications occurred during hospitalization including pneumothorax (20.6%), hypertension (20.4%), cannula dysfunction (20.2%), seizure (14.9%), renal failure requiring hemofiltration (14.7%), infectious complications (10.3%), thrombi (7.4%), intracranial hemorrhage or infarction (6.6%), hemolysis (5.3%), cannula site bleeding (4.4%), gastrointestinal bleeding (3.7%), oxygenator failure (2.8%), other bleeding events (2.8%), brain death (1.9%), and myocardial stun (0.9%) (Table 4).

**Table 4:** Outcomes and the incidence of complications of VV ECMO in the treatment of neonatal respiratory failure

Outcome	Number of studies reporting outcome	summary point estimate (CI 95%)
Hospital mortality		
Pooled mortality	4 (347)	12% (5–18%)
Mortality during ECMO	2 (179)	10% (0.8–19.2%)
Mortality after decannulation	2 (179)	6.1% (2.6–9.6%)
Medical complications		
Gastrointestinal bleeding	1 (107)	3.7% (0.1–7.3%)
Intracranial hemorrage/infarction	3 (293)	6.6% (3.7–9.4%)
Cannula site bleeding	2 (179)	4.4% (−1.8–10.6%)
Hemolysis	1 (114)	5.3% (1.2–9.4%)
Other bleeding events	1 (107)	2.8% (−0.3–5.9%)
Seizure	2 (161)	14.9% (9.4–20.4%)
Brain death	1 (107)	1.9% (−0.7–4.4%)
Pneumothorax	1 (107)	20.6% (12.9–28.2%)
Hypertension	1 (54)	20.4% (9.6–31.1%)
Myocardiac stun	1 (114)	0.9% (−0.8–2.6%)
Renal failure needing hemofiltration	3 (275)	14.7% (5.9–23.5%)
Infectious complications	1 (107)	10.3% (4.5–16%)
Thrombi	1 (54)	7.4% (0.4–14.4%)
Mechanical complications		
Oxygenator failure	1 (72)	2.8% (−1.0–6.6%)
Cannula failure	2 (126)	20.2% (−4.2–44.7%)

*VV ECMO* Veno-venous extracorporeal membrane oxygenation

### Subgroup analysis

**Fig. 3:**
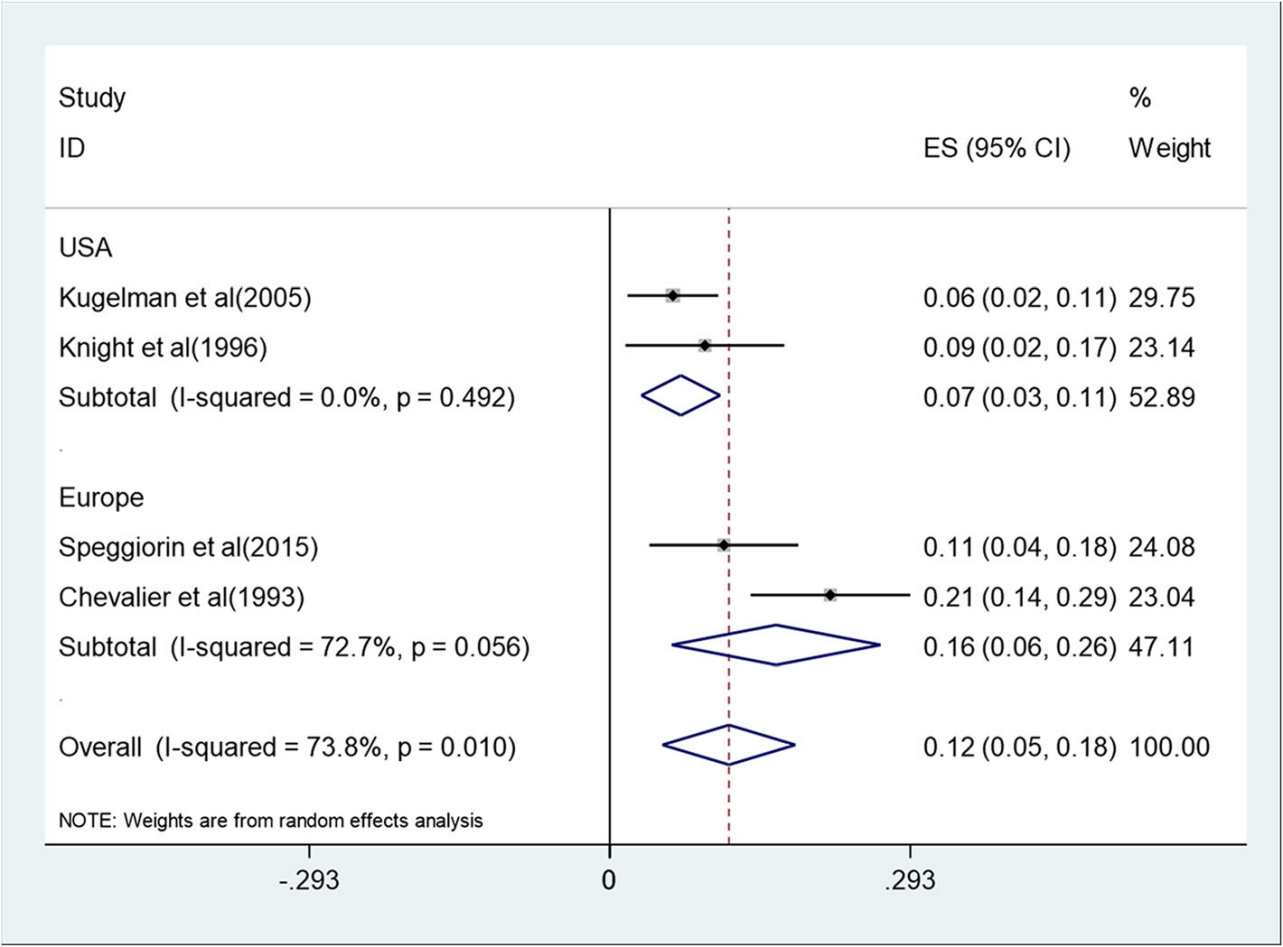
Forest plot of mortality across racial groups

**Fig. 4:**
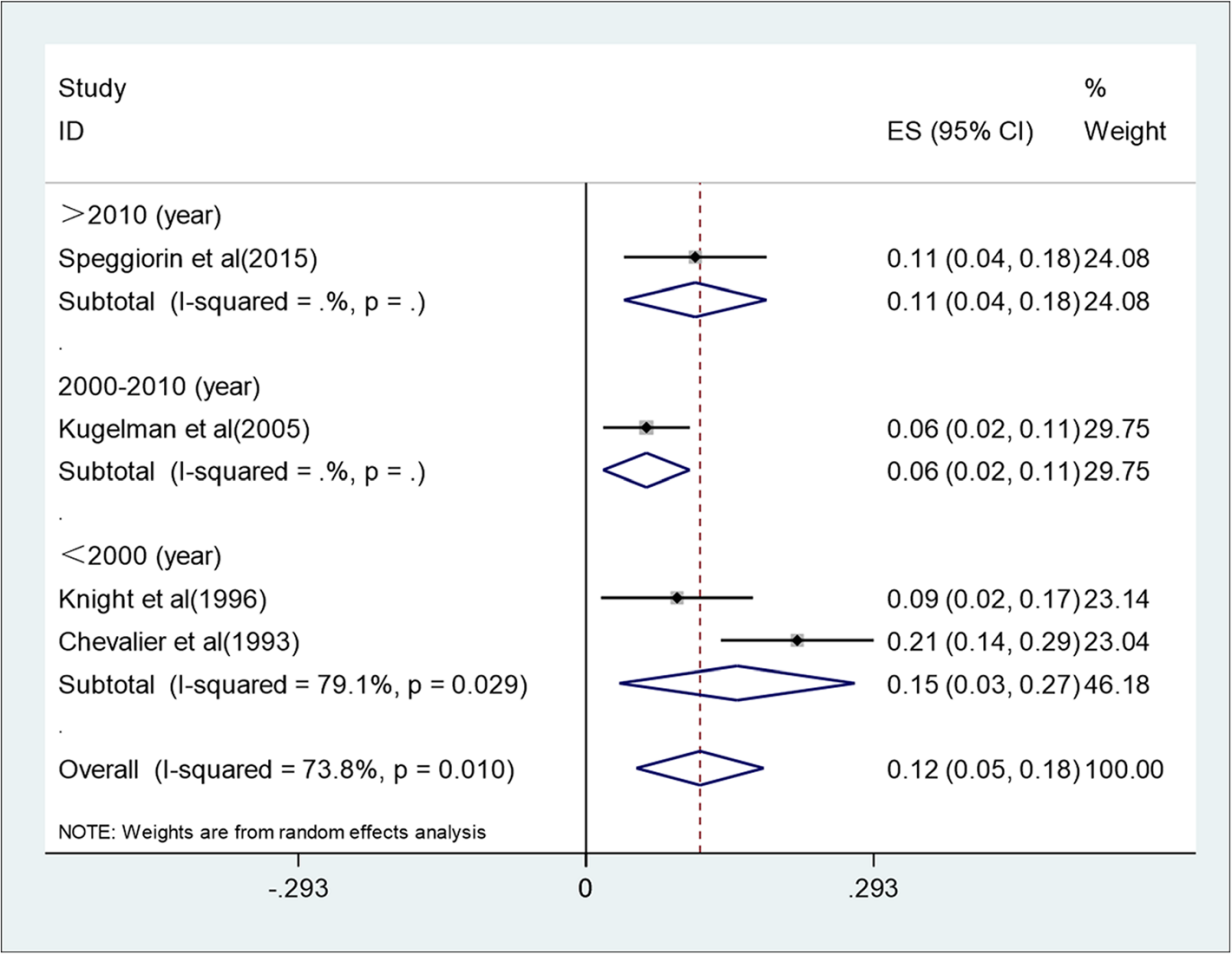
Forest plot of mortality from different publication years

**Fig. 5:**
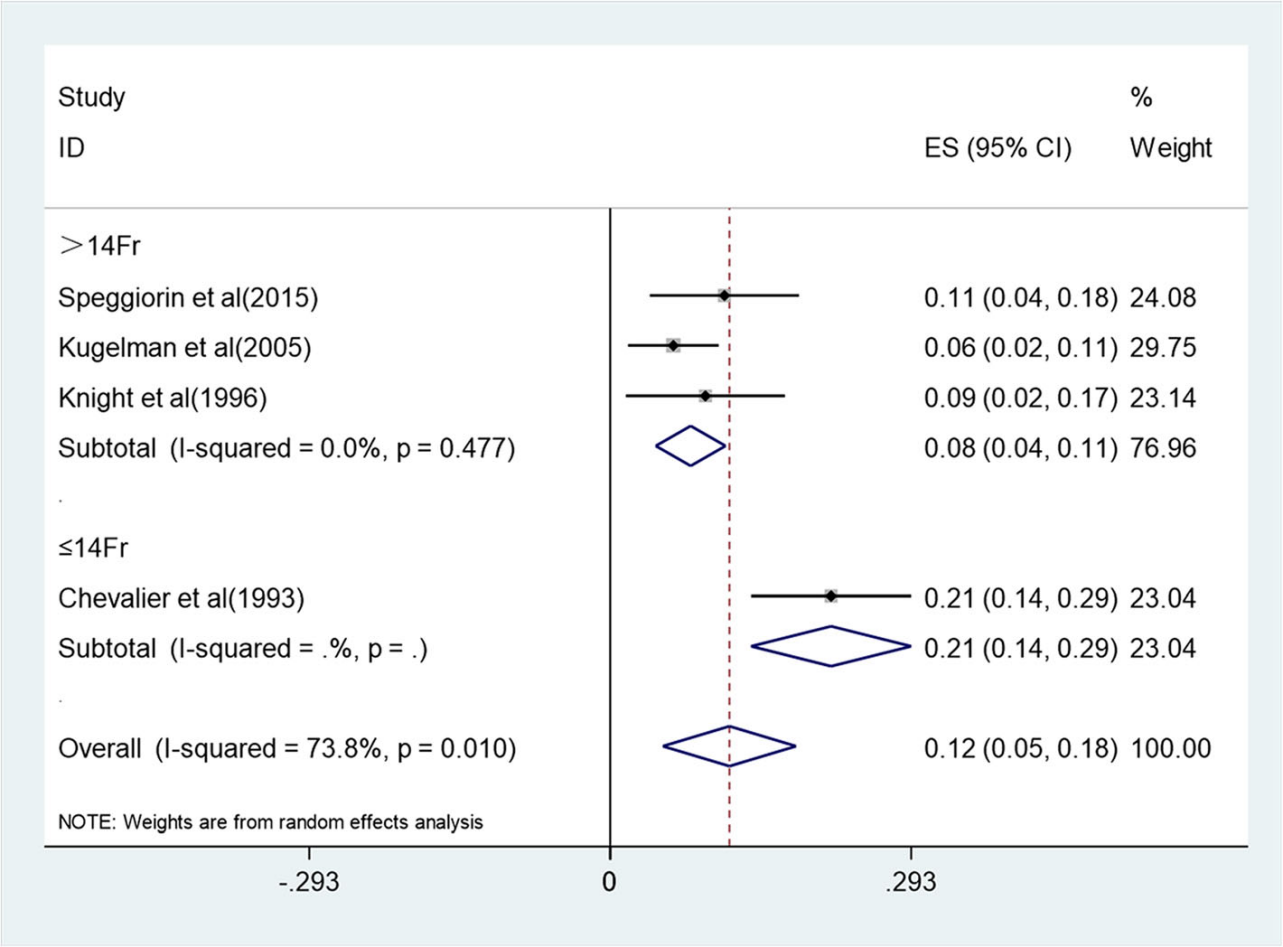
Forest plot of mortality with different maximum cannula sizes

**Fig. 6:**
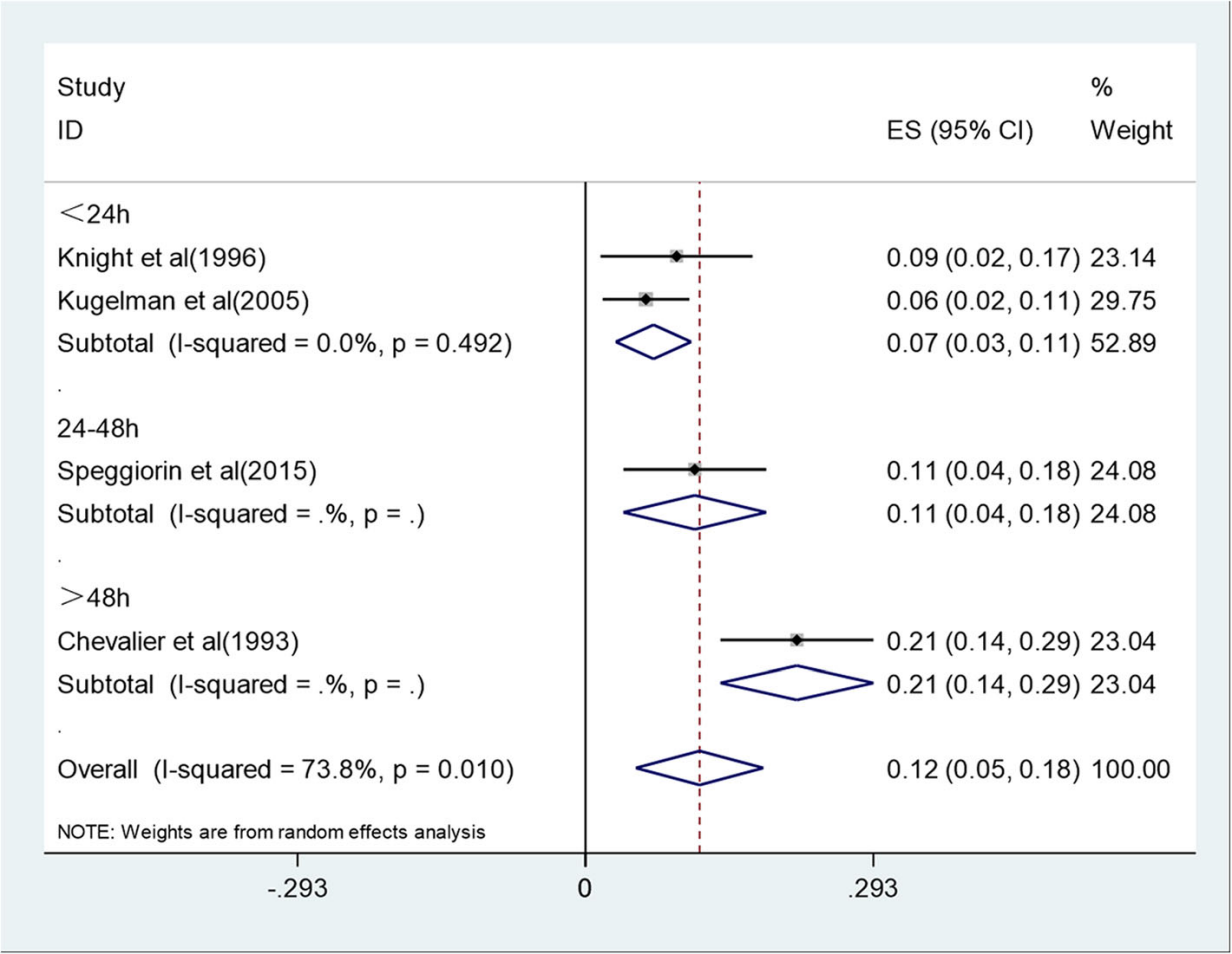
Forest plot of mortality at different ages at the beginning of ECMO

Racial group, publication year, maximum cannula size, and age at the beginning of ECMO might be sources of heterogeneity between studies. So we performed subgroup analysis from these four aspects (Fig. 3, Fig. 4, Fig. 5, Fig. 6). The results showed that maximum cannula size and age at the beginning of ECMO were sources of heterogeneity between studies, while racial group and publication year were not sources of heterogeneity between studies. Besides, the heterogeneity between studies might also originate in disease severity, ECMO equipment type, medical center’s level, the experience of the medical staff who operates ECMO, and some other factors.

Because the included studies are fewer, we didn’t perform meta-regression analysis and publication bias.

## Discussion

Our study showed that the survival rate of neonates with respiratory failure after receiving VV ECMO was 88%, higher than that (73%) of neonates with respiratory failure treated by ECMO according to ELSO registry report in January 2019 [21]. The reason might be that the data of ELSO come from the mixed population of VA ECMO and VV ECMO, and most patients who receive VA ECMO have hemodynamic instability and need cardiac support, thus reduce the survival rate. According to the ELSO database, the survival rate of VA ECMO for neonatal respiratory failure between 2012 and 2017 was 70%, while that of VV ECMO was 80% [22].

Our results also showed that mortality rate of neonates in the Kugdman et al.’s study was lowest [18], while that in the Chevalier et al.’s study was highest [19]. According to the ELSO database, neonates with MAS have the highest survival rate, followed by neonates with PPHN and CDH [6]. On one hand, neonates with MAS enrolled in the Kugdman et al.’s study might have more stable respiratory status, plus new treatment modalities (NO, HFV, PS) were used and the ECMO team was more experienced at that time, thus improve the survival rate. On the other hand, in the Chevalier et al.’s study, cannula applied on neonates was small, indicating that this group of neonates were small, and ECMO equipment was not advanced at the early time, all these factors might result in the relatively high mortality of this study.

An overall survival rate of 88% was seen in the 347 neonates, higher than that of other age groups by VV perfusion according to the ELSO database. Actually, different age groups have different disease spectrum. For neonatal ECMO, the most common diagnoses are CDH, MAS, and PPHN, accounting for almost 75% of all neonatal respiratory ECMO cases [22]. Whilst for pediatric ECMO and adult ECMO, the most common diagnoses are pneumonia and acute respiratory distress syndrome (ARDS) [6]. Prognosis of neonates with MAS, RDS and PPHN is promising due to good response to supplemental therapies such PS and iNO. In contrast, no studies have shown the beneficial effects of surfactant for adult and pediatric ARDS, which may explain the lower survival rate of pediatric and adult ECMO for respiratory failure caused by ARDS and pneumonia. In 2017, the international ARDS collaborative group provided the first consensus definition for neonatal ARDS [23]. However, the above studies of neonatal ECMO were performed in the pre-ARDS era, in which ARDS was usually considered as neonatal RDS. Actually, ARDS and RDS are two significant different diseases with different reactions to surfactant, and they should be diagnosed and treated independently. Besides, mortality rate is also associated with other factors such as annual hospital ECMO volume for neonates and adults, but not for pediatric cases [24].

In our study, complications including mechanical complications, bleeding, hypertension, seizure, and renal failure occurred during hospitalization. According to the ELSO, the most common complication of neonatal ECMO for respiratory failure is mechanical complication, such as clots in the ECMO circuit [6], which is consistent with our study results. Bleeding and clots complications are multifactorial. Even though an ideal test of anticoagulation for patients is lacking, continuous unfractionated heparin and close monitoring of anticoagulation are required to reduce the risk of thrombosis and hemorrhage [25]. In our study, the rates of neurologic complications such as intracranial hemorrhage (ICH)/infarction and seizure are high as well, with 6.6 and 14.9%, respectively. When analyzing the ELSO registry report in 2016, neonates using ECMO have the highest rate of neurologic complications, with an ICH incidence of around 7.6% [6]. Various pre-existing factors like low birth weight, acidosis, hypoxia, hypotension, and organ failure have been found to be associated with neurologic injury. Besides, some ECMO factors such as modality of ECMO, hemorrhage, seizures, and development of new organ failure increase the risk of central neural system injuries further [26]. Therefore, understanding of risk factors associated with neonates and knowing how to deal with them are important to reduce complications. With the evolving indications for ECMO and the dramatically changed monitoring technology and supportive therapies over these years, the outcomes of patients have been improved greatly. Further attempts, such as by improving the equipment of ECMO, are needed to determine whether such events can be reduced.

Since a double-lumen catheter was designed in 1989, VV ECMO has been increasingly used in neonatal respiratory failure [27, 28]. VV ECMO has a few advantages over VA ECMO. During VV ECMO, ligation of the carotid arteries is avoided, pulmonary circulation and coronary artery perfusion are maintained well, thus reduce the left ventricular afterload. Studies have showed that VV ECMO compared favorably to VA ECMO for cardiovascular support [29, 30]. Some previous studies have also shown that VV ECMO was associated with lower rates of neurologic complications as compared with VA ECMO [27, 31, 32].

In this study, to minimize potential bias of observational study, we established inclusion and exclusion criteria strictly to provide accurate prevalence and incidence estimation, and we limited the minimum sample size of each study to 50 to reduce publication bias. Moreover, we excluded the studies published in the ELSO database to avoid data duplication and reduce selection bias, because only the selected medical centers have the chance to register in the ELSO database, which will increase selection bias. Therefore, detailed VV ECMO data of other medical centers outside the ELSO database was collected in this study.

### Limitations

There are some limitations in our study. Firstly, all the studies were non-RCT studies, which increased the risk of bias. Statistic quality of systematic review and meta-analysis is best assessed by RCTs. However, a pure randomized study is rare, whereas accurate studies are relatively common and provide most of the available evidence [33]. Secondly, only studies written in English were included, which might cause language bias. Thirdly, less than 10 studies were included, and publication bias and meta regression analysis were not performed, which might pose a potential risk of publication bias. Fourthly, the number of included studies was small and there was moderate heterogeneity among the studies. Fifthly, Some data in the original study could not be obtained, such as pump type and membrane type, and the baseline standards of each study might be inconsistent, many potential factors might play a role in our analysis. Lastly, the inclusion criteria might also result in the omission of potentially important studies, such as case reports and small sample studies. However, small sample studies might be affected by publication bias, historical bias, selective reporting, and other methodological deficiencies, which increase the risk of bias.

## Conclusions

The results of this study showed that although VV ECMO treatment for neonatal respiratory failure might lead to some complications including pneumothorax, hypertension, cannula dysfunction, seizure, renal failure and so on, the survival rate during hospitalization is still high. Larger samples and higher quality of randomized controlled studies are needed to provide a more reliable basis for the application of VV ECMO in neonates with respiratory failure.

## Data Availability

The data supporting our findings can be found by contacting us (400702@hospital.cqmu.edu.cn).

## Abbreviations

ARDS: Acute respiratory distress syndrome
CDH: Congenital diaphragmatic hernia
CI: Confidential interval
ELSO: Extracorporeal life support organization
HFOV: High-frequency oscillatory ventilation
iNO: Inhaled nitric oxide
ICH: Intracranial hemorrhage
MOOSE: Meta-analysis of observational studies in epidemiology
MAS: Meconium aspiration syndrome
NOS: NEWCASTLE-Ottawa Scale
PPHN: Persistent pulmonary hypertension of newborn
RDS: Respiratory distress syndrome
PS: Pulmonary surfactant
PRISMA: Preferred reporting items for systematic review and meta-analysis
RCTs: Randomized controlled trials
VV ECMO: Venovenous extracorporeal membrane oxygenation
VA ECMO: Venoarterial extracorporeal membrane oxygenation
